# The association of serum uric acid level and the uric acid to creatinine ratio with non alcoholic fatty liver disease in children with obesity

**DOI:** 10.1038/s41598-025-26023-y

**Published:** 2025-11-26

**Authors:** Hanife Ayşegül Arsoy, Özlem Kara

**Affiliations:** 1 Department of Pediatric Gastroenterology, Hepatology and Nutrition, University of Health Sciences Bursa, Yuksek Ihtisas Training and Research Hospital, Bursa, Turkey; 2 Department of Pediatric Endocrinology, University of Health Sciences Bursa, Yuksek Ihtisas Training and Research Hospital, Bursa, Turkey; 3 Department of Pediatric Gastroenterology, Hepatology and Nutrition, University of Health Sciences Bursa, Bursa City Hospital, Doğanköy, Doğanköy İç Yolu, Bursa, 16110 Ni̇lüfer/Bursa Turkey

**Keywords:** Childhood obesity, Non-alcoholic fatty liver disease, Serum uric acid, Serum uric acid/creatinine ratio, Biomarkers, Endocrinology, Gastroenterology

## Abstract

It has been demonstrated that elevated serum uric acid (sUA) levels elicit both pro-inflammatory and pro-oxidative effects. A growing body of evidence suggests that this may play a contributory role in the development of Non-alcoholic fatty liver disease (NAFLD) in children with obesity. The objective of the present study is to evaluate the association between sUA levels, the sUA/creatinine (Cr) ratio and paediatric NAFLD. This single-center, cross-sectional, comparative study was conducted at a tertiary care center. The study cohort comprised 228 patients with obesity (body mass index (BMI) ≥ 95th percentile) and 167 healthy controls without obesity or overweight. Both groups were 9–18 years of ages, matched for sex and pubertal stage. A diagnosis of NAFLD was made following an ultrasound examination of the liver, with other possible causes of hepatic disease being excluded. A significantly elevated level of sUA, and sUA/Cr values weres observed in the group of patients with obesity in comparison to the control group (*p* < 0.001). In the group with obesity, NAFLD was detected by abdominal ultrasonography in 169 (74.2%) patients, whereas NAFLD was not detected in 59 (25.8%) patients. The levels of sUA and sUA/Cr were significantly elevated in the obese NAFLD group in comparison to the obese non-NAFLD and control groups (*p* < 0.001). An elevated WC, ALT, and the ratio of sUA/Cr were associated with an increased risk of NAFLD. A one-unit increase in sUA/Cr was found to be associated with an increased risk of NAFLD (OR = 1.323, 95% CI: 1.001–1.748). The findings of our study indicate that an elevated WC, ALT, and the ratio of sUA/Cr were associated with an increased risk of NAFLD. Nevertheless, no such correlation was identified between sUA and NAFLD.

## Introduction

Non-alcoholic fatty liver disease (NAFLD) represents the most common form of liver disease observed in paediatric patients, with a notable increase in its occurrence in parallel with the rising prevalence of obesity in developed countries^1^. The definition of NAFLD is a condition characterised by an excess of fat accumulation in the liver, observed through histological or radiological means, exceeding 5% of the total liver volume. This accumulation occurs in the absence of significant alcohol consumption. It has been demonstrated that children with excess body weight, including those who are obese, are at an increased risk of developing NAFLD. Additionally, they are prone to developing cardiometabolic risk factors such as insulin resistance, prediabetes, diabetes, dyslipidaemia, and central adiposity. Furthermore, certain racial and ethnic groups are predisposed to an increased risk of developing NAFLD^1^. Although the data available are limited, it has been noted that patients diagnosed with NAFLD in childhood have higher morbidity and mortality rates than those diagnosed in adulthood^2^.

Uric acid represents a metabolic product of purine nucleotides, typically acquired by humans through elevated intake of purine-rich foods (e.g., meat and seafood), monosaccharide fructose and alcoholic beverages^3^. Fructose has been demonstrated to stimulate the activity of adenosine monophosphate (AMP) deaminase in hepatocytes, resulting in substrate-dependent phosphate depletion (adenosine triphosphate (ATP) depletion). This, in turn, has been shown to increase uric acid production^4^. Elevated serum uric acid (sUA) levels have been demonstrated to elicit pro-inflammatory and pro-oxidative effects^5,6^. Both ATP depletion and increased sUA have been demonstrated to cause hepatocellular damage and to play a role in the progression of NAFLD^7^. Hyperuricaemia has been identified as an independent risk factor for the degree of liver damage in patients with NAFLD^8,9^.

Uric acid is primarily excreted through the renal system; as a consequence, sUA levels are influenced by renal function^10^. Furthermore, insulin resistance elevates sUA levels by reducing renal excretion of uric acid^11^. A recent study demonstrated that a sUA/Cr is significantly releated with NAFLD in the general population. As a result, renal function should be carefully monitored in patients with NAFLD^12^. Accordingly, in the current study, the sUA/Cr ratio was utilised for the purpose of standardising the sUA level in paediatric NAFLD patients, in a manner consistent with the approach previously employed in adult studies.

With the increase in obesity, NAFLD is a public health problem that is increasing rapidly in children and its prevalence has doubled in the last 20 years. Given the potential for chronic liver disease, cirrhosis and related complications to develop in children, it is imperative that they are detected and prevented at the earliest opportunity through lifestyle changes. The aim of the present study is to evaluate the association of paediatric NAFLD with sUA levels and the sUA/Cr ratio.

## Materials and methods

### Study design and subjects

This comparative, single-centre, cross-sectional study was conducted from January 2021 to June 2021 in the paediatric gastroenterology and paediatric endocrinology unit of a referral care centre. The study cohort consisted of 228 patients with obesity (body mass index (BMI) ≥ 95th percentile) and 167 healthy controls with normal BMI, matched for age, sex and pubertal stage. Participants were aged between 9 and 18 years in both groups. The patients with other known diseases, as well as those with endocrine and liver diseases, were excluded from the study. The BMI standard deviation (SD), weight SD and height SD were calculated using the age- and sex-specific reference values^13^. High blood pressure was described as systolic or diastolic blood pressure above the 95th percentile for a given age, sex and height^14^. The following biochemical parameters were analysed: aspartate aminotransferase (AST), alanine aminotransferase (ALT), alkaline phosphatase (ALP), gamma-glutamyl transferase (GGT), fasting glucose (FG), fasting insulin, ferritin, triglyceride (TG), and total cholesterol (TC). The levels of high-density lipoprotein cholesterol (HDL-C), low-density lipoprotein cholesterol (LDL-C), thyroid stimulating hormone (TSH), free T4 and sUA were analysed and a ratio was calculated on the basis of the levels of sUA and creatinine. In all patients whose ALT value (upper limit value: 22U/L for girls, 26U/L for boys) was ≥ 2 of the upper limit for three months and ALT > 80 U/L at the time of screening, causes of liver disease such as autoimmune or viral hepatitis, alpha-1 antitrypsin deficiency, Wilson’s disease, drug usage, and celiac disease were excluded^1,15^.

All study participants underwent abdominal ultrasonography (USG) to look for the presence of steatosis. Hepatic steatosis detected on abdominal USG was classified as NAFLD. The group with obesity was divided into two subgroups, one with and one without non-NAFLD, according to ultrasound measurements and the anthropometric, laboratory, and radiological data of these groups were in comparison with the data from the healthy control group.

Prior to the start of the study, participants were properly informed of the nature and objectives of the study and formal informed consent was obtained. The study was approved by the local ethics committee and conducted per the Declaration of Helsinki of 1975 (2011-KAEK-25 2020/12 − 02).

### Anthropometric and blood pressure measurements

Measurements of height, weight, waist circumference (WC) and blood pressure (BP) were carried out by an expert paediatric nurse. The weights of all participants (clothed in light indoor attire and unshod) were measured using a balance beam scale (SECA) to the nearest 0.1 kg. The participants’ heights (barefoot) were measured using a portable height measurer to the nearest 0.1 cm. BMI was calculated by dividing weight by the square of height. Waist circumference was measured using a flexible measuring tape at a level halfway between the lower rib margin line and the iliac crest, to the nearest 0.5 cm.

Blood pressure was measured in the morning after 10 min of quiet sitting. Participants were seated with their arm supported at heart level. BP was measured using an automatic OMRON M6 monitor with the appropriate cuff size.

### Laboratory analysis

Biochemical parameters AST, ALT, GGT, ALP, sUA, Cr were analysed spectrophotometrically using Abbott ARCHITECT plus kits. Following a 12-hour fast, FG, TC, TG, HDL-C, LDL-C and insulin were determined. Serum ferritin was analysed by the CMIA chemiluminescent microparticle immunoassay using the Abbott ARCHITECT plus instrument. Serum glucose and lipid concentrations were analysed using standardised methods on the Cobas 8000 analyser. The Cobas 8000” was used to measure insulin using the standard method. Serum TSH, sT4 were measured by electrochemulusens method. A fasting glucose level between 100 and 125 mg/dL was defined as impaired fasting glucose, and a level ≥ 126 mg/dL was defined as having overt diabetes^16^. The Homeostasis Model Assessment for Insulin Resistance (HOMA-IR) index was used to assess insulin resistance^17^. HOMA-IR formula (fasting blood glucose (mg/dl) x fasting insulin (µIU/ml) / 405). In lipid profile, TG and HDL-C cut-off values were based on TG ≥ 150 mg/dL and HDL-C < 40 mg/dL^18^. SUA/Cr was calculated as sUA divided by serum creatinine. GFR was calculated with Schwartz formula [k X height (cm)/SCr (mg/ dl); k factor was 0.55, 0.55 and 0.7 for children, female adolescents and male adolescents, respectively].

### Abdominal ultrasound

All patients enrolled in the study underwent abdominal USG using a Hitachi Hi-Vision Preirus machine by radiologists who were experienced in pediatric patients and blinded to laboratory results but not to anthropometric features, as body habitus is apparent during scanning. Fatty liver was graded on a scale as grade 0 (no steatosis, normal liver echogenicity pattern), grade 1 (mild steatosis, mild and diffuse parenchymal enhancement with normal visualisation of diaphragm and portal margins), grade 2 (moderate steatosis, moderate and diffuse increase in fine echoes with slight deterioration of visualisation of portal vein margins and diaphragm) and grade 3 (severe steatosis and significant increase in fine echoes with weak or no visualisation of portal vein margins and diaphragm)^19^.

### Statistical analysis

The data was examined using the Shapiro-Wilk test to determine whether or not it presents a normal distribution. The results were presented as mean ± standard deviation or frequency and percentage. Normally distributed data were compared with independent samples t-test or one-way ANOVA. Kruskal Wallis and Mann Whitney U tests were used for nonnormally distributed data. The Bonferroni test was used as a multiple comparison test. Categorical variables were compared using Pearson chi-square tests between groups. Binary Logistic Regression was performed, and the crude odds ratios (OR) and their 95% Confidence Intervals (CIs) were reported. Multivariate binary logistic regression analysis was performed, and the adjusted ORs and 95% CIs were obtained. Power analysis was performed with G*Power 3.1 statistical program and the total sample size was determined with Power (1-β) = 0.8 and α = 0.05. For all analyses, the significance level was set at *p* < 0.05.

Statistical analyses were performed with IBM SPSS 29.0.2.0 (IBM Corp. Released 2023. IBM SPSS Statistics for Windows, Version 29.0.2.0 Armonk, NY: IBM Corp.)

## Results

### Anthropometric, clinical and laboratory parameters of the children with obesity and controls

A total of 228 patients with obesity, and 167 healthy children as a control group, were included in the study. The age (both groups were 9–18 years, [mean 14.17 ± 2.41 for children with obesity group, mean 14.15 ± 2.67 for control group]), sex and puberty distribution of the study and control groups were similar (*p* > 0.05). The data indicated that the anthropometric variables, systolic and diastolic blood pressure, and the variables pertaining to BMI exhibited statistically significant differences between the study groups (*p* < 0.001), with the obese group displaying higher levels than the control group. A significantly elevated level of fasting insulin, HOMA-IR, AST, ALT, GGT, TC, TG, LDL-C, ferritin, sUA, and sUA/Cr values was observed in the group of patients with obesity in comparison to the control group (*p* < 0.001). Conversely, a markedly diminished level of HDL-C was evident in the patient group. No statistically significant difference was observed in Cr and GFR values between the groups (*p* > 0.05). The data concerning the anthropometric, clinical and laboratory parameters of the two groups are presented in Table 1.

**Table 1:** Anthropometric, clinical, and laboratory parameters according to the groups.

	Obese group *n* = 228	Control group *n* = 167	*p*
Age (years)	14.17 ± 2.41	14.15 ± 2.67	0.920^‡^
Gender (Female)	143 (63)	94 (56)	0.197^¶^
Pubertal	212 (93)	148 (89)	0.132^¶^
Weight sd	2.73 ± 1.23	-0.22 ± 0.81	**< 0.001†**
Height sd	0.37 ± 1.18	-0.02 ± 0.92	**0.009†**
BMI (kg/m^2^)	31.12 ± 5.56	19.84 ± 2.53	**< 0.001†**
BMI sd	2.52 ± 0.79	-0.23 ± 0.77	**< 0.001†**
Waist circumference (cm)	101.91 ± 15.14	69.34 ± 7.08	**< 0.001** ^‡^
SBP sd	0.72 ± 0.95	0.11 ± 0.88	**< 0.001†**
DBP sd	0.86 ± 0.88	0.47 ± 0.78	**< 0.001†**
Fasting glucose (mg/dl)	87.96 ± 7.99	85.78 ± 7.39	**0.016†**
Fasting insulin (µU/L)	30.99 ± 19.74	11.35 ± 6.71	**< 0.001†**
HOMA-IR	6.84 ± 4.80	2.42 ± 1.55	**< 0.001†**
AST (U/L)	22.54 ± 14.89	18.01 ± 4.96	**0.009†**
ALT (U/L)	30.43 ± 30.50	13.17 ± 6.03	**< 0.001†**
GGT (U/L)	19.45 ± 12.17	11.65 ± 6.16	**< 0.001†**
Total cholesterol (mg/dl)	162.31 ± 31.29	144.48 ± 29.76	**< 0.001†**
Triglycerides (mg/dl)	139.43 ± 69.66	78.72 ± 30.24	**< 0.001†**
HDL-cholesterol (mg/dl)	44.36 ± 10.56	55.61 ± 12.48	**< 0.001†**
LDL-cholesterol (mg/dl)	91.38 ± 28.21	75.15 ± 23.65	**< 0.001†**
Ferritin (ng/ml)	44.10 ± 29.70	29.36 ± 20.28	**< 0.001†**
Serum uric acid (mg/dl)	5.32 ± 1.43	4.14 ± 1.03	**< 0.001†**
Serum creatinine (mg/dl)	0.63 ± 0.12	0.62 ± 0.12	0.583^‡^
sUA/Cr	8.64 ± 2.39	6.81 ± 1.76	**< 0.001†**
GFR	160.43 ± 37.75	160.20 ± 33.18	0.565**†**

NAFLD: Non-alcoholic fatty liver disease, sd: standard deviation; BMI: Body mass index; SBP: Systolic blood pressure; DBP: Diastolic blood pressure; HOMA-IR: homeostasis model assessment for insulin resistance; AST: aspartate aminotransferase; ALT: alanine aminotransferase; GGT: gamma glutamyltransferase; HDL: high-density lipoprotein; LDL: low-density lipoprotein; sUA/Cr: Serum Uric acid/Creatinin; GFR: glomerular filtration rate.

Descriptive statistics are reported as mean ± standard deviation and frequency with percentage (%).

^¶^Pearson Chi-square test **†**Mann-Whitney U test ^‡^Independent samples t-test.

### Anthropometric and clinical parameters according to the NAFLD/ non-NAFLD **detected by USG in obese and control groups**

In the group with obesity, NAFLD was detected by abdominal ultrasonography in 169 (74.2%) patients, whereas NAFLD was not detected in 59 (25.8%) patients. The study population was stratified into two groups according to their NAFLD status, namely a NAFLD group and a non-NAFLD group. The data from these two groups were then compared with that of a control group. The non-NAFLD obese group demonstrated a markedly elevated prevalence of female patients in comparison to the other groups (*p* = 0.004). The data on body weight SD, height SD, BMI, BMI SD, WC, SBP SD and DBP SD demonstrated significant discrepancies between the groups, particularly in the obese NAFLD group (*p* < 0.001). Table 2 presents the anthropometric and clinical laboratory parameters observed in the groups.

**Table 2:** Anthropometric and clinical parameters according to the NAFLD/ non-NAFLD detected by USG in obese and control groups.

	Obese NAFLD group *n* = 169	Obese Non-NAFLD group *n* = 59	Control group *n* = 167	*p*
Age (years)	14.09 ± 2.35	14.41 ± 2.59	14.15 ± 2.67	0.559**†**
Gender (Female)	96 (57)^a^	47 (80)^b^	94 (56)^a^	**0.004** ^**¶**^
Pubertal	159 (94)	53 (90)	148 (89)	0.197^¶^
Body weight sd	2.89 ± 1.27^a^	2.26 ± 0.98^a^	-0.22 ± 0.81^b^	**< 0.001†**
Height sd	0.40 ± 1.22^a^	0.27 ± 1.07^ab^	-0.02 ± 0.92^b^	**< 0.001** ^**‡**^
BMI (kg/m^2^)	32.14 ± 5.78 ^a^	28.18 ± 3.49 ^a^	19.84 ± 2.53 ^b^	**< 0.001†**
BMI sd	2.65 ± 0.83 ^a^	2.16 ± 0.61 ^a^	-0.23 ± 0.77 ^b^	**< 0.001†**
Waist circumference (cm)	104.81 ± 15.12^a^	93.60 ± 11.84^b^	69.34 ± 7.08^c^	**< 0.001** ^**‡**^
SBP sd	0.83 ± 097 ^a^	0.41 ± 0.80 ^b^	0.11 ± 0.88 ^b^	**< 0.001†**
DBP sd	0.98 ± 0.88 ^a^	0.55 ± 0.78 ^b^	0.47 ± 0.78 ^b^	**< 0.001†**

NAFLD: Non-alcoholic fatty liver disease; sd: standard deviation; BMI: Body mass index; SBP: Systolic blood pressure; DBP: Diastolic blood pressure.

Descriptive statistics were reported as mean ± standard deviation and frequency with percentage (%).

^¶^Pearson Chi-square test **†**Kruskal-Wallis Test ^‡^One-Way ANOVA.

^abc^The Bonferroni test was used for pairwise comparisons. The “a”, “b” and “c” superscripts show the results of pairwise comparisons between groups; values with unlike letters were significantly different among groups.

### Laboratory parameters according to the NAFLD/ non-NAFLD detected by USG in obese and control groups

**Fig. 1:**
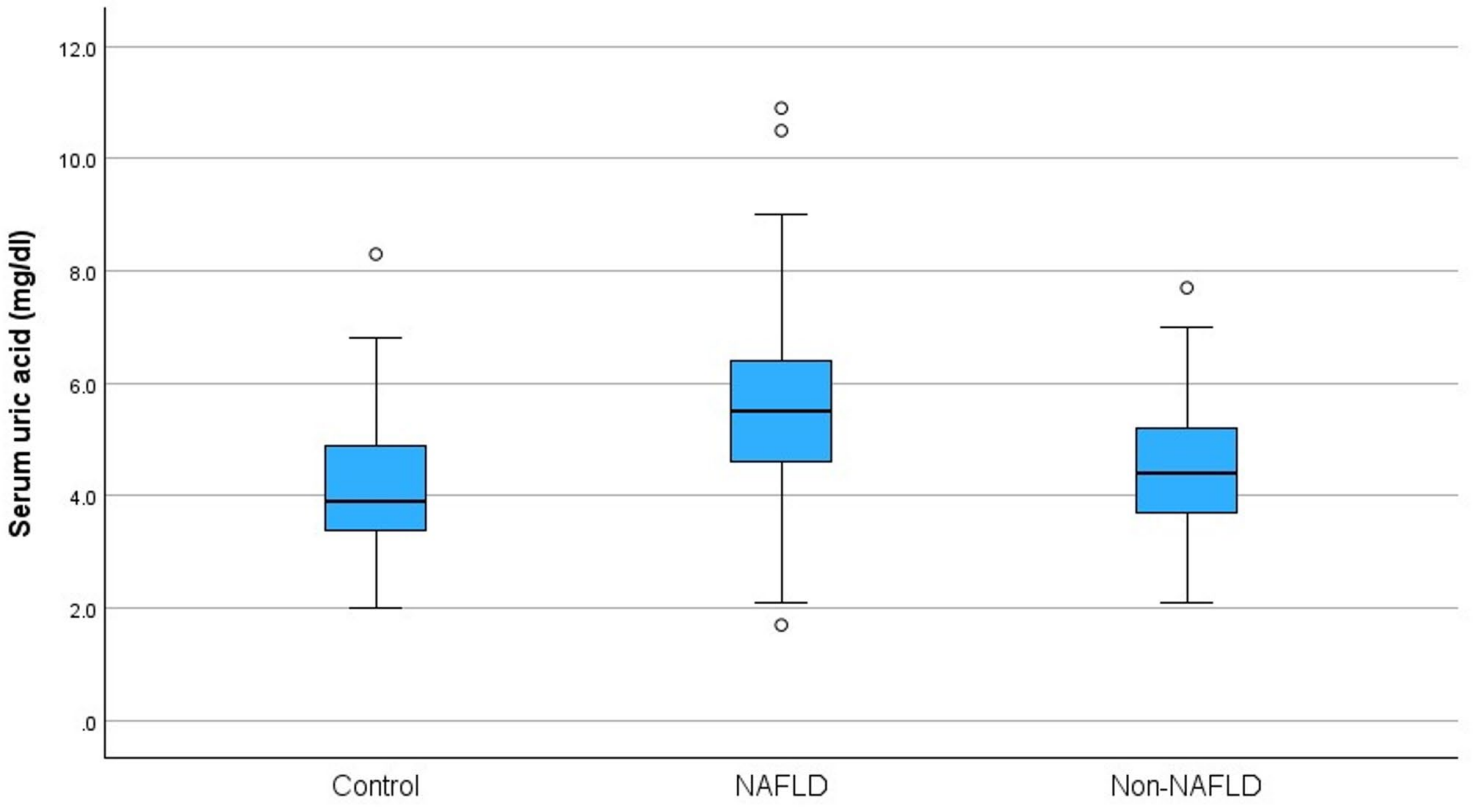
Comparison of sUA levels between the groups.

**Fig. 2:**
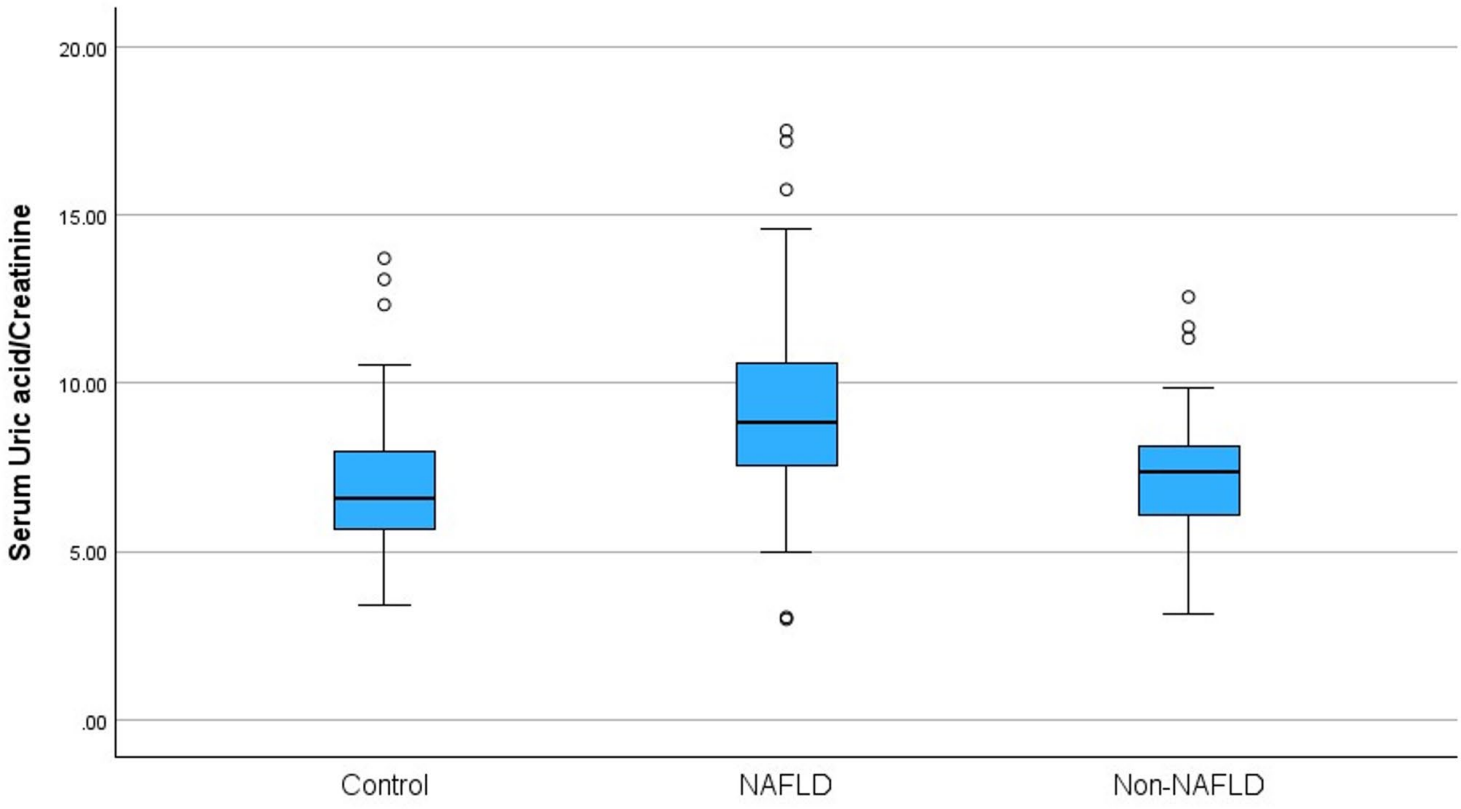
Comparison of sUA/Cr ratios between the groups.

Fasting insulin, HOMA-IR, ALT, AST, GGT, ferritin, sUA, sUA/Cr were notably higher in the obese NAFLD group compared to the obese non-NAFLD and control groups (*p* < 0.001). HDL-C levels were also significantly lower in the obese NAFLD group compared to the other two groups (*p* < 0.001). It is noteworthy that the sUA level was 5.59 ± 1.44 mg/dl in the obese NAFLD group, which exhibited a statistically significant difference (*p* < 0.001) when compared to the obese non-NAFLD and control groups. No significant difference was found between the sUA levels of obese non-NAFLD and control group, respectively (4.52 ± 1.10^b^ vs. 4.14 ± 1.03^b^ mg/dl). Once more, it is noteworthy that the sUA/Cr ratio was 9.11 ± 2.39 in the obese NAFLD group, when compared to the obese non-NAFLD and control groups (*p* < 0.001). No significant difference was found between the sUA/Cr ratios of obese non-NAFLD and control group, respectively (7.28 ± 1.18^b^ vs. 6.81 ± 1.76^b^). Comparison of sUA levels and sUA/Cr ratios between the groups are given in Figs. 1 and 2. No significant difference was found in serum Cr levels between the groups. Laboratory parameters of the groups are given in Table 3.

**Table 3:** Laboratory parameters according to the NAFLD/ non-NAFLD detected by USG in obese and control groups.

	Obese NAFLD group *n* = 169	Obese Non-NAFLD group *n* = 59	Control group *n* = 167	*p*
Fasting glucose (mg/dl)	88.36 ± 8.27^a^	86.83 ± 7.08^ab^	85.78 ± 7.39^b^	**0.039**
Fasting insulin (µU/L)	33.72 ± 21.20^a^	23.17 ± 11.83^b^	11.35 ± 6.71^c^	**< 0.001**
HOMA-IR	7.49 ± 5.21^a^	4.99 ± 4.96^b^	2.42 ± 1.55^c^	**< 0.001**
AST (U/L)	24.80 ± 16.47^a^	16.03 ± 4.85^b^	18.01 ± 4.96^c^	**< 0.001**
ALT (U/L)	35.82 ± 33.63^a^	14.98 ± 6.07^b^	13.17 ± 6.03^b^	**< 0.001**
GGT (U/L)	21.03 ± 13.14^a^	14.92 ± 7.16^b^	11.69 ± 6.16^c^	**< 0.001**
Total cholesterol (mg/dl)	160.72 ± 29.33^a^	166.86 ± 36.22^a^	144.41 ± 29.76^b^	**< 0.001**
Triglycerides (mg/dl)	145.89 ± 74.49^a^	120.90 ± 49.48^a^	78.72 ± 30.24^b^	**< 0.001**
HDL-cholesterol (mg/dl)	42.86 ± 10.18^a^	48.68 ± 10.51^b^	55.61 ± 12.48^c^	**< 0.001**
LDL-cholesterol (mg/dl)	90.97 ± 26.29^a^	92.56 ± 33.35^a^	75.15 ± 23.63^b^	**< 0.001**
Ferritin (ng/ml)	47.97 ± 31.58^a^	33.06 ± 19.90^b^	29.36 ± 20.28^b^	**< 0.001**
Serum uric acid (mg/dl)	5.59 ± 1.44^a^	4.52 ± 1.10^b^	4.14 ± 1.03^b^	**< 0.001**
Serum creatinine (mg/dl)	0.62 ± 0.13	0.63 ± 0.11	0.62 ± 0.12	0.801
sUA/Cr	9.11 ± 2.39^a^	7.28 ± 1.18^b^	6.81 ± 1.76^b^	**< 0.001**
GFR	164.0.65 ± 39.20^a^	150.03 ± 31.26^b^	160.20 ± 33.18^ab^	**0.032**

NAFLD: Non-alcoholic fatty liver disease; HOMA-IR: homeostasis model assessment for insulin resistance; AST: aspartate aminotransferase; ALT: alanine aminotransferase; GGT: gamma-glutamyltransferase; HDL: high-density lipoprotein; LDL: low-density lipoprotein; sUA/Cr: Serum Uric acid/Creatinin; GFR: glomerular filtration rate.

Descriptive statistics were reported as mean **±** standard deviation.

Kruskal-Wallis Test was used.

^abc^The Bonferroni test was used for pairwise comparisons. The “a”, “b” and “c” superscripts show the results of pairwise comparisons between groups; values with unlike letters were significantly different among groups.

### Logistic regression of variables related to the risk of NAFLD

Age, gender, sUA, sUA/Cr, WC, fasting insulin, HOMA-IR, AST, ALT variables were analysed by univariate logistic regression analysis to determine the factors associated with NAFLD (Table 4). As a result, while the variables other than age were found statistically significant, only the significant variables were included in the multivariate binary logistic regression analysis. The results of the multivariate binary logistic regression analysis indicated that WC, ALT and sUA/Cr were significant variables. An elevated WC, ALT, and ratio of sUA/Cr are associated with an increased risk of NAFLD (Table 4). The study revealed that a one-unit increase in waist circumference was associated with an increased risk of NAFLD (OR = 1.044-fold, 95% CI: 1.013–1.076). Similarly, a one-unit increase in ALT level was linked to an elevated risk of NAFLD (OR = 1.081-fold, 95% CI: 1.016–1.149). Additionally, a one-unit increase in sUA/Cr level was associated with an increased risk of NAFLD (OR = 1.323-fold, 95% CI: 1.001–1.748).

**Table 4:** Logistic regression of variables related to the NAFLD.

	Univariate				Multivariate			
	*p*	OR	OR 95% Cl		*p*	OR	OR 95% Cl	
			Lower	Upper			Lower	Upper
Gender (Male)	0.002	2.978	1.474	6.017	-	-	-	-
Age	0.387	0.946	0.835	1.072	-	-	-	-
Waist circumference	< 0.001	1.058	1.033	1.084	0.005	1.044	1.013	1.076
Fasting insulin	< 0.001	1.052	1.023	1.081	-	-	-	-
HOMA-IR	< 0.001	1.241	1.101	1.399	-	-	-	-
AST	< 0.001	1.156	1.084	1,232	-	-	-	-
ALT	< 0.001	1.142	1.085	1.203	0.014	1.081	1.016	1.149
Triglycerides	0.016	1.007	1.001	1.013	-	-	-	-
Ferritin	0.001	1.022	1.008	0.035	-	-	-	-
Serum uric acid	< 0.001	1.964	1.480	2.605	-	-	-	-
sUA/Cr	< 0.001	1.710	1.503	1.946	0.049	1.323	1.001	1.748

OR: Odds ratio; CI: Confidence Interval; NAFLD: Non-alcoholic fatty liver disease; HOMA-IR: homeostasis model assessment for insulin resistance; AST: aspartate aminotransferase; ALT: alanine aminotransferase; sUA/Cr: Serum Uric acid/Creatinin.

## Discussion

With the sharp increase in obesity in the last 20 years, the prevalence of NAFLD in the paediatric population has increased and has been shown to reach %3–10^20^. In children and adolescents, the prevalence of histologically confirmed NAFLD in liver autopsy samples was 9.6%, increasing to 38% in obese children^21^. In paediatric NAFLD, liver biopsy for screening is not practical or feasible due to the wide clinical spectrum and increased incidence of the disease, it being an invasive procedure and ethical concerns about its necessity. Therefore, the need for non-invasive tests in screening, diagnosis and management is increasing. In this current study, we aim to demonstrate the association of sUA levels and sUA/Cr ratio with paediatric NAFLD, in addition to laboratory parameters known to be associated with NAFLD.

The results of our study demonstrated that the levels of sUA were markedly elevated in the cohort of obese patients with NAFLD in comparison to both the obese non-NAFLD group and the control group. It is noteworthy that no statistically significant difference was observed in the sUA levels between the obese non-NAFLD group and the control group. This result indicates the possibility that a high sUA level may be linked to the development of NAFLD. In a recently published study, similar to our study, significantly higher sUA levels were found in the overweight/obesity group with NAFLD, detected by ultrasonography, compared to the overweight/obesity group without NAFLD^22^. The study included a substantial number of children with overweight or obesity; nevertheless, a control group comprising healthy children was not included in the study design^22^. In a study comparing histologically proven NAFLD in paediatric patients with obese and lean control groups, it was shown that basal sUA levels were higher in patients with histologically proven NAFLD than in obese and lean control groups^23^. In adults, there are cross-sectional and prospective studies examining the relationship between sUA level and NAFLD and demonstrating that sUA is an independent risk factor for NAFLD; these studies used ultrasonographic diagnosis as the basis for the diagnosis of NAFLD^24,25^. A recent study conducted on adults diagnosed with NAFLD by USG revealed a correlation between an elevated sUA level and the frequency of hyperuricaemia, and an increased frequency and severity of fatty liver disease^26^. A meta-analysis of twenty-five studies demonstrated that the risk of NAFLD in patients with hyperuricaemia increased 1.97 times for every 1 increase in sUA level, pooled OR of 1.97 (95% CI, 1.69–2.29)^27^. In our study, when the association with NAFLD was considered together with other parameters, we demonstrated that an increase of one unit in sUA level was associated with a 1.96-fold increased risk of NAFLD, with an OR of 1.964 (95% CI, 1.480–2.605). However, when confounding factors were excluded from the analysis, it was found that sUA level alone had no effect on the risk of NAFLD.

The results of studies published in the last five years indicate a positive correlation between the sUA/sCr ratio and NAFLD^12,28–31^. A recent study identified a positive relationship between NAFLD and sUA/sCr. When sUA/sCr increased by 1, the risk in the mild NAFLD group increased by 1.147 times (OR = 1.147, 95% CI: 1.099–1.196; *p* < 0.001) and the risk of the moderate-to-severe NAFLD group increased by 1.275 times (OR = 1.275, 95% CI: 1.212–1.341;*p* < 0.001)^26^. In an adult study in which NAFLD was diagnosed by controlled attenuation parameter (CAP) value of ≥ 274 dB/m detected by Fibroscan, the sUA/Cr ratio was positively associated with CAP values and the prevalence of NAFLD. In that study, when the highest sUA/Cr (Q4; 7.60–27.60) group increased by 1, the risk of NAFLD was increased by 1.80 times (OR = 1.80, 95% CI: 1.24–2.62, *p* = 0.0019)^28^. In another cross-sectional study in which the diagnosis of NAFLD was made by abdominal computed tomography, it was found that the sUA/SCr ratio was significantly higher in patients with NAFLD compared to the non-NAFLD group. In the same study, an increase of one unit in OR of sUA/Cr was associated with a 1.209 times increased risk of NAFLD (OR = 1.209, 95% CI: 1.101–1.326, *p* < 0.0001)^29^. In our study, when the association with NAFLD was considered together with other parameters, there was a statistically significant increase in sUA/sCr, which was found to increase the risk of NAFLD by 1.710 times (OR = 1.710, 95% CI: 1.503–1.946, *p* < 0.001). After the confounding factors had been excluded, it was found that a one-unit increase in the sUA/Cr level was associated with a 1.323 times increased risk of NAFLD (95% CI: 1.001–1.748). The sUA lost significance in the multivariate model, while the sUA/Cr ratio remained associated with pediatric NAFLD (*p* = 0.049, OR = 1.323). Although this borderline result should be interpreted cautiously, it suggests that kidney function–adjusted uric acid may be a more specific marker. Similar associations have been reported in both pediatric and adult populations^30–32^. Taken together, these findings indicate that sUA/Cr may be more informative than sUA alone, but confirmation in larger pediatric cohorts is required. In the near future, studies focusing on this data in the paediatric age group will be able to access a more extensive range of information.

Waist circumference is an effective parameter for measuring intra-abdominal fat volume or area and for assessing abdominal obesity^33,34^. A recent study has demonstrated that WC is the most significant risk factor for predicting NAFLD among body composition indicators in adults^35^. It is abdominal obesity, rather than the general concept of obesity, that is a contributor to liver fibrosis in children with NAFLD. In addition, in these children WC is the only component of the metabolic syndrome associated with liver fibrosis^36^. In our study, we found that a one-unit increase in WC was an independent component that increased the risk of NAFLD by OR = 1.044 times (95% CI: 1.013–1.076).

The European Society for Pediatric Gastroenterology, Hepatology and Nutrition (ESPGHAN) guidelines recommend both ALT and USG for NAFLD screening in children^21^. In the North American Society for Pediatric Gastroenterology, Hepatology and Nutrition (NASPGHAN) guidelines, initial screening with ALT is recommended in obese/overweight children (with risk factors)^1^. However, it has been reported that significant histological changes, including advanced fibrosis, can occur in children with NAFLD, even in those with only mildly elevated ALT levels^37^. AASLD guidelines states that the use of ALT alone as a screening test may underestimate the extent of liver damage^15^. Despite the lack of clarity surrounding the ALT cut-off values associated with NAFLD, our study has demonstrated that a one-unit increase in ALT, an established biomarker, is associated with an increased risk of NAFLD, with an odds ratio of 1.081 (95% CI: 1.016–1.149).

There are some limitations of our study. It is of particular importance to note that the study comprised patients attending outpatient clinics for paediatric gastroenterology and paediatric endocrinology at our tertiary care centre; as a result, the findings may not be representative of the general paediatric population. As our study was a cross-sectional one, we were able to ascertain the clinical situation prevailing in the current time period. Another limitation of our study is that the diagnosis of pediatric NAFLD relied only on ultrasonography. Although USG is widely used because it is non-invasive and feasible in children, its sensitivity for mild steatosis is limited, and it may occasionally lead to under- or overestimation^38–40^. Nevertheless, as recommended by pediatric NAFLD guidelines, USG remains the most practical tool in clinical and research settings, and our results still contribute valuable data in this field. Another limitation of our study is that intra- and inter-observer variability in ultrasonographic grading of steatosis was not formally evaluated, which may affect reproducibility. The last limitation of our study is that dietary fructose intake, an established contributor to uric acid metabolism and NAFLD risk, was not assessed in our study.

## Conclusion

The results of our study suggest that an elevated sUA/Cr ratio, increased waist circumference and elevated ALT levels are independent risk factors for the development of NAFLD in children. The sUA/Cr ratio, derived from routine and inexpensive laboratory tests, may serve as a supportive biomarker for pediatric NAFLD, particularly where advanced diagnostics are limited. Its value is likely greatest when combined with ALT and anthropometric measures such as BMI or waist circumference. A positive association between NAFLD and sUA level as well as sUA/Cr ratio was identified through logistic regression analysis. It is imperative that further studies be conducted within the paediatric age group to evaluate this association.

## Data Availability

The datasets generated and analysed during the current study are not publicly available due patient data comes from the hospital’s electronic medical record system but are available from the corresponding author on reasonable request.
